# Editorial: Global excellence in gene and cell therapy: Europe

**DOI:** 10.3389/fmed.2023.1154360

**Published:** 2023-02-21

**Authors:** Liliana Mendonça, Johannes Boltze, Clévio Nóbrega

**Affiliations:** ^1^Center for Neuroscience and Cell Biology (CNC), University of Coimbra, Coimbra, Portugal; ^2^Institute of Interdisciplinary Research, University of Coimbra, Coimbra, Portugal; ^3^Department of Chemistry and Pharmacy, Faculty of Sciences and Technology, University of Algarve, Faro, Portugal; ^4^School of Life Sciences, University of Warwick, Coventry, United Kingdom; ^5^Algarve Biomedical Center Research Institute (ABC-RI), University of Algarve, Faro, Portugal; ^6^Faculty of Medicine and Biomedical Sciences, University of Algarve, Faro, Portugal

**Keywords:** regulatory landscape for ATMPs, AAVs-mediated gene delivery, riboswitch-regulated AAVs, regulatory T cells production and regulation, AAV8-mediated gene therapy combined with chemotherapy

Gene therapy changes protein expression in cells through the insertion of genes and/or gene expression-modulating tools, including gene editing. It can be used in the treatment of a variety of diseases, including genetic disorders, by reducing the levels of disease-causing proteins or by increasing the expression of missing or protective proteins. Cell therapy by the transplantation of cells or tissues aims to repair, regenerate, or replace a specific cell population and/or tissue to reestablish the physiological function performed by the cells/tissues to replace. In this line, *Global Excellence in the Gene and Cell Therapy: Europe* Research Topic was launched with the goal of highlighting the latest advancements in Gene and Cell Therapy field and reflecting on the future challenges faced by researchers across Europe.

Six papers were accepted and published for this Research Topic, from which three describe original research data, one is a systematic review, one is a narrative paper, and the last one is a perspective article.

The systematic review by Au et al., *Gene Therapy Advances: A Meta-Analysis of AAV Usage in Clinical Settings* addressed the use of Adeno-associated viruses (AAVs) as gene delivery vehicles to drive long-term transgene expression. This meta-analysis of two decades of AAV application in gene therapy, in 136 clinical trials, provided an up-to-date overview of the use of AAVs in clinical trials, while evaluating the major challenges in the AAVs' translatability into clinical practice. These comprise restricted gene packaging capacities, off-target transduction, and immunogenicity. The authors also pointed out some aspects in this research field that should be further investigated, namely the development of optimal dosing regimens based on dose-dependent toxicity and efficacy studies, the engineering of new capsid variants to improve transduction and reduce immunogenicity, and the use of better-suited promoters. Accordingly, this paper provides an overview of AAV-mediated gene therapy.

In their original research paper *COL17A1 editing via homology-directed repair in junctional epidermolysis bullosa*, Petković et al. reported a gene editing approach to correct a frameshift mutation within the *COL17A1* gene associated with junctional epidermolysis bullosa (JEB). In this work, type XVII collagen (C17)-deficient JEB keratinocytes were edited with either Cas9 nuclease or nickase (Cas9n) ribonucleoproteins (RNP) and a single-stranded oligonucleotide (ssODN) homology-directed repair (HDR) template. Next-generation sequencing of RNP-nucleofected keratinocytes demonstrated an HDR efficiency of approximately 38% with the high-fidelity Cas9 nuclease, a mutation-specific sgRNA, and a ssODN template. Moreover, gene-corrected JEB keratinocytes upon differentiation into skin equivalents displayed enhanced adhesive strength to laminin-332 and an accurate deposition of C17 along the basement membrane zone. Overall, this work describes a promising CRISPR/Cas9-based gene editing strategy.

Kujala et al. report in their original research paper *AAV8-mediated sVEGFR2 and sVEGFR3 gene therapy combined with chemotherapy reduces the growth and microvasculature of human ovarian cancer and prolongs the survival in mice* the antiangiogenic and antitumoral effects on ovarian cancer (OVCA) of adeno-associated virus 8 (AAV8)-mediated expression of soluble VEGF receptors (sVEGFRs) sVEGFR2 and sVEGFR3 combined with the chemotherapy drugs paclitaxel and carboplatin. Mice inoculated with human OVCA cells were tested with: (i) AAV8-CMV and chemotherapy, (ii) AAV8-sVEGFR2, (iii) AAV8-sVEGFR3, (iv) AAV8-sVEGFR2 and AAV8-sVEGFR3, and (v) AAV8-sVEGFR2 and AAV8-sVEGFR3 with chemotherapy. Authors reported that the therapeutic combination of AAV8-sVEGFR2 and AAV8-sVEGFR3 with chemotherapy significantly limited intratumoral angiogenesis and tumor growth. Therefore, this work demonstrates the potential synergistic benefits of combining antiangiogenic therapy and chemotherapy in human OVCA treatment.

The original research work by Eriksson et al. *Optimized riboswitch-regulated AAV vector for VEGF-B gene therapy* investigated a method to temporally regulate therapeutic gene expression with Riboswitches. Several tetracycline and toyocamycin-inducible ON-riboswitches were tested. The tetracycline-dependent K19 riboswitch displayed the best expression control of several transgenes. The gene expression induction was 6- to 10-fold, reversible, dose-dependent, and took place within hours upon tetracycline administration. The authors further optimized the gene cassette to control the expression of VEGF-B, a gene with therapeutic potential for cardiovascular diseases. They found that the riboswitch function was promoter-independent, the use of two or three riboswitches simultaneously reduced leakiness and improved the temporal dynamic range, and a linker sequence between the riboswitches improves their functionality. Therefore, this study indicates that Riboswitch-regulated transgene expression is a promising strategy for safe, dose-controlled gene therapy and provides important insights into the optimization of this strategy.

The review *Barriers to Treg therapy in Europe: from production to regulation* (Hennessy et al.) analyzes some critical aspects in the development of advanced therapy medicinal products (ATMPs) such as regulatory T cells (Tregs). Several parameters required to ensure the safety, efficacy, and quality of these medicinal products are discussed, namely the source of cells to be employed, the methods through which the cells are isolated and expanded, and the cells' storage. Regulatory issues are also analyzed including the mandatory compliance with Good Manufacturing Procedure (GMP) guidelines and the challenges of its implementation. Moreover, an overview of the currently approved ATMPs in Europe and the costs of the development of this type of medicinal products is provided.

Salazar-Fontana contributed with the perspective article *A Regulatory Risk-Based Approach to ATMP/CGT Development: Integrating Scientific Challenges With Current Regulatory Expectations*, providing an overview on the European Union and United States Regulatory Landscape for ATMP/Cell and Gene Therapy (CGT) Products. Some aspects of the ATMP/CGT development and the guidelines established by the Food and Drug Administration (FDA) and the European Medicines Agency (EMA) on the quality, non-clinical and clinical considerations were reviewed. The paper discusses, among other subjects, the timelines for product development and the review of the license application. Thus, the accelerated drug development processes Orphan Drug Designation (ODD), EU PRIME, and US RMAT were considered. Moreover, the EMA and FDA's different approach regarding the regulatory requirements of Genetically Modified Organisms (GMO) for ATMP/ CGT was also addressed.

Overall, the articles in the present Research Topic advanced on the development of new gene delivery and gene editing tools and provided an overview of the Regulatory Landscape for ATMPs.

## Author contributions

LM wrote the manuscript. JB and CN revised the manuscript. All authors approved the submitted version.

